# The prevalence and clinical outcome of supraventricular tachycardia in different etiologies of pulmonary hypertension

**DOI:** 10.1371/journal.pone.0245752

**Published:** 2021-01-20

**Authors:** Zdenka Fingrova, David Ambroz, Pavel Jansa, Jan Kuchar, Jaroslav Lindner, Jan Kunstyr, Michael Aschermann, Ales Linhart, Stepan Havranek

**Affiliations:** 1 1^st^Faculty of Medicine, 2^nd^ Department of Medicine-Department of Cardiovascular Medicine, General University Hospital, Charles University, Prague, Czech Republic; 2 Regional Hospital in Tabor, Tabor, Czech Republic; 3 1^st^ Faculty of Medicine, 2^nd^ Department of Surgery–Department of Cardiovascular Surgery, General University Hospital, Charles University, Prague, Czech Republic; 4 1^st^ Faculty of Medicine, Department of Anaesthesiology, Resuscitation and Intensive Medicine, General University Hospital, Charles University, Prague, Czech Republic; Fondazione Toscana Gabriele Monasterio & Scuola Superiore Sant'Anna, Pisa, Italy, ITALY

## Abstract

**Purpose:**

Patients with pulmonary hypertension (PH) frequently suffer from supraventricular tachycardias (SVT). The main purpose of our study was to identify the cumulative incidence of SVT in patients with different etiologies of PH. The secondary objective was to analyse the clinical impact of SVT.

**Methods:**

We retrospectively studied the prevalence of SVT and the clinical outcome in 755 patients (41% males; 60 ± 15 years; mean follow-up 3.8 ± 2.8 years) with PH of different etiologies. The prevalence of SVT was analysed separately in isolated pre-capillary PH (Ipc-PH) and in patients with combined post- and pre-capillary PH (Cpc-PH).

**Results:**

The prevalence of SVT in the Ipc-PH group (n = 641) was 25% (n = 162). The most prevalent arrhythmias were atrial fibrillation followed by a typical atrial flutter (17% and 4.4% of all Icp-PH patients). An excessive prevalence of SVT was found in patients with pulmonary arterial hypertension associated with congenital heart disease (35%, p = 0.01). Out of the overall study population, Cpc-PH was present in 114 (15%) patients. Patients with Cpc-PH manifested a higher prevalence of SVT than subjects with Ipc-PH (58; 51% vs. 162; 25%; p <0.0001) and were more likely to have persistent or permanent atrial fibrillation (38; 29% vs. 61; 10%; p <0.0001). Parameters significantly associated with mortality in a multivariate analysis included age, male gender, functional exercise capacity and right atrial diameter (p < 0.05). Neither diagnosis of SVT nor type of arrhythmia predicted mortality.

**Conclusions:**

The study detected a significant prevalence of SVT in the population of PH of different origins. Different spectrum and prevalence of arrhythmia might be expected in different etiologies of PH. Patients with an elevated post-capillary pressure showed a higher arrhythmia prevalence, predominantly due to an excessive number of atrial fibrillations. The diagnosis of SVT was not associated with mortality.

## Introduction

Pulmonary hypertension (PH) develops in multiple clinical conditions. PH can be categorized according to clinical presentation, pathological findings and haemodynamic characteristics [1,2]. In recent years, various treatment strategies have been established which have improved haemodynamics, exercise capacity, and quality of life [1]. Despite those advances, the prognosis of PH is generally inauspicious.

Supraventricular tachycardias (SVT), including atrial fibrillation (AF), have been reported as a common condition in patients with idiopathic pulmonary arterial hypertension (PAH) [3,4] (range of cumulative incidence 10–36%), including all types of PAH [4–8], Eisenmenger`s syndrome [9] or inoperable chronic thromboembolic pulmonary hypertension (CTEPH) [4,5,7]. Recently, high prevalence of AF and atrial tachycardia (AT) in CTEPH patients treated with pulmonary endarterectomy was identified with an excessive number of newly diagnosed AF/AT during long-term follow-up after surgery [10]. It has also been demonstrated that SVT in patients with PH frequently lead to clinical deterioration [3–5,7,11]. Therefore, it is not surprising that SVT development has also been investigated as a predictor of mortality [3–5,12]. A strategy attempting sinus rhythm (SR) restoration in patients with idiopathic PH appears to improve the clinical outcome [3,5].

Rottlaender et al. has shown that the prevalence of AF in PH patients could differ according to the etiology of PH [11].

However, the epidemiology of SVT in different etiological PH groups is not well documented. Little is known about the current clinical outcome of SVT in PH patients in this modern era of arrhythmia treatment. Therefore, we analysed data from a large single-centre database aiming to identify the prevalence and impact of SVT in patients with different classes of PH. To further evaluate the role of different hemodynamic profiles on SVT epidemiology in PH patients, we have compared patients with isolated pre-capillary PH (Ipc-PH) and those with combined post- and pre-capillary PH (Cpc-PH). The secondary objective was to describe the differences between the two groups in terms of predisposing factors and evaluate the impact of SVT on the clinical outcome.

## Methods

We performed an analysis of a dedicated registry of consecutive patients, who were diagnosed and treated for PH at a single centre between 2003 and 2017. The final follow-up was set to December 2018. The study was performed according to the principles of good clinical practice and in compliance with the Declaration of Helsinki. The whole study was approved by the local Ethics committee (Ethics Committee in General University Hospital in Prague, No. 1121/16-S-IV). All patients gave a written informed consent agreeing to data collection and analysis for scientific purposes.

The protocol used in this study has been previously described in detail [8]. In brief, all study patients underwent a routine baseline work-up according to contemporary standards [1,13,14] including all indicated noninvasive and invasive methods and right heart catheterization to confirm and classify PH. The diagnosis of PH required confirmation of the pulmonary artery mean pressure (PAMP) ≥ 25 mm Hg by the baseline right heart catheterization.

Patients with isolated post-capillary PH (defined as PAMP ≥ 25 mm Hg and pulmonary artery wedge pressure (PAWP) > 15 mm Hg but having a pulmonary vascular resistance (PVR) ≤ 3 Wood Units (WU)) were excluded [1]. In addition, we also excluded patients with CTEPH which were indicated for a pulmonary endarterectomy in whom the surgery plays a dominant role modifying their risk of arrhythmia development. Patients with Ipc-PH were required to have a resting PAWP ≤ 15 mm Hg. This cohort was compared to patients with Cpc-PH defined as subjects with PAMP ≥ 25 mm Hg and simultaneous elevation of PAWP > 15 mm Hg and PVR > 3 WU.

All patients were seen regularly at 1 to 6 monthly intervals, or whenever clinically indicated. For further evaluation, the time of the PH diagnosis was set as the beginning of the study. Routine examinations and standard 12-leads ECG were obtained as part of the regular follow-up program. A 24-hour, 48-hour or longer ECG monitoring was performed when arrhythmia was clinically suspected. The period of monitoring was driven by the clinical situation. A prevalent SVT was defined as evidence of the presence of a documented SVT on the standard 12-lead ECGs and/or ECG monitors in a patient’s personal history or at the time of diagnosis or during a follow-up. The diagnosis of an SVT was confirmed by an experienced cardiologist in every case. A detected arrhythmia was classified as a SVT, according to current guidelines [15], as tachycardia (atrial and/or ventricular rates more than 100 bpm at rest) with the mechanism of which involves tissue from the His bundle or above. For analysis purposes, a SVT was classified as follows: Cavo-tricuspid isthmus-dependent (typical and reverse typical) atrial flutter (AFL), paroxysmal, persistent or permanent AF, atrioventricular nodal reentrant tachycardia (AVNRT), atrioventricular reentrant tachycardia (AVRT), focal AT and finally “other AT” including all types of SVT of an unclear origin. Management of SVT in our centre was performed according to current guidelines [16,17].

### Statistical analysis

Continuous variables were expressed as means with standard deviations. After testing for normality (Shapiro-Wilk's test) data was compared using the 2-tailed t-test for independent samples or advanced ANOVA tests to compare more than two means. Categorical variables were expressed as percentages and compared by the χ2–test or the Kruskal-Wallis test when appropriate. The Gehan-Wilcoxon test was applied to compare mortality rates in univariate analysis. Cox proportional hazard regression analysis was used to stratify predictors of mortality. P-value < 0.05 was considered as significant. All analyses were performed using the STATISTICA vers.12 software (Statsoft, Inc., Tulsa, USA).

## Results

### Baseline characteristics of the cohort

A total of 755 patients (mean age 60 ± 15 years; 41% males) were included in the analysis. Baseline clinical and demographical characteristics of the total population and subgroups are shown in Table 1. The spectrum of patients according to clinical classification of PH is in Table 2. Patients with Cpc-PH in general were slightly older, more prevalently had diabetes mellitus, had a reduced six-minute walking test (6MWT) distance, higher left atrial (LA) diameter, slightly bigger end-diastolic left ventricular diameter, reduced tricuspid annular plane systolic excursion (TAPSE), a more elevated PAMP and right atrial pressure (RAP), a higher occurrence of advanced NYHA class, and a higher mortality rate, than patients with Ipc-PH. Out of all Cpc-PH patients, 9 subjects with a suspected significant left heart disease were excluded. In the final Cpc-PH group (n = 114), the mean PAWP was 19 ± 2 mm Hg; range 16–25 mm Hg; mode of 16 mm Hg; median of 18 mm Hg; interquartile range (IQR) of 17–20 mm Hg.

**Table 1:** Baseline demographic and clinical characteristics.

	Total n = 755	Ipc-PH n = 641	Cpc-PH n = 114	p value
**Age (years)**	60 ± 15	59 ± 15	62 ± 14	0.03
**Males**	307 (41%)	266 (41%)	41 (36%)	NS
**Art. Hypertension**	431 (57%)	356 (56%)	75 (62%)	NS
**Stroke/Systemic embolism**	56 (7%)	43 (7%)	13 (11%)	NS
**Diabetes mellitus**	215 (28%)	172 (27%)	43 (38%)	0.02
**Specific therapy**	475 (61%)	404 (63%)	71 (62%)	NS
**NYHA (class)**				
• **I**	7 (1%)	6 (1%)	1 (1%)	NS
• **II**	138 (18%)	125 (20%)	13 (11%)	0.023
• **III**	500 (66%)	417 (65%)	83 (73%)	NS
• **IV**	107 (14%)	90 (14%)	17 (15%)	NS
• **III and IV**	607 (70%)	507 (79%)	100 (88%)	0.026
**6MWT (meters)**	321 ± 122	325 ± 123	298 ± 111	0.047
**LA in PLAX (mm)**	41 ± 8	41 ± 7	45 ± 8	< 0.0001
**LV EF (%)**	63 ± 8	63 ± 8	68 ± 8	NS
**LVEDD in PLAX (mm)**	46 ± 8	45 ± 8	47 ± 7	0.02
**RA in A4C (mm)**	47 ± 11	47 ± 11	48 ± 11	NS
**RV in A4C (mm)**	44 ± 10	44 ± 10	45 ± 10	NS
**TAPSE (mm)**	18 ± 5	19 ± 5	17 ± 5	0.02
**PAMP (mmHg)**	48 ± 16	47 ± 16	52 ± 15	0.003
**PAWP (mmHg)**	12 ± 5	10 ± 3	19 ± 2	< 0.0001
**RAP (mmHg)**	10 ± 6	9 ± 5	15 ± 6	< 0.0001
**Follow-up (years)**	3.8 ± 2.8	3.8 ± 2.8	3.7 ± 2.6	NS
**Dead**	415 (55%)	341 (53%)	74 (65%)	0.02
**SVT prevalence**	220 (29%)	162 (25%)	58 (51%)	< 0.0001

Values are expressed as mean ± standard deviation or as n (%). NS–non-significant. Ipc-PH–isolated pre-capillary pulmonary hypertension; Cpc-PH–combined post- and pre-capillary pulmonary hypertension; PH–pulmonary hypertension; 6MWT–six minute walking test; LA–left atrium; LV–left ventricle; EF–ejection fraction; LVEDD–left ventricular end-diastolic diameter; RA–right atrium; RV–right ventricle; TAPSE–tricuspid annular plane systolic excursion; PAMP–pulmonary arterial mean pressure; PAWP–pulmonary arterial wedge pressure; RAP–right atrial pressure; SVT–supraventricular tachycardia; PLAX–parasternal long axis view; A4C –apical four chamber view.

**Table 2:** Spectrum of patients with Ipc-PH according to clinical classification.

Clinical classification	Ipc-PH n = 641
Group 1 PH–PAH	
• Idiopathic/Heritable	220 (34%)
• associated with CTD	62 (10%)
• associated with CHD	52 (8%)
Group 3 PH–Lung disease/Hypoxia	140 (22%)
Group 4 PH–CTEPH	130 (20%)
Group 5 PH–Unclear and/or multifactorial mechanism	37 (6%)

Values are expressed as n (%). NS–non-significant. Ipc-PH–isolated pre-capillary pulmonary hypertension; PH–pulmonary hypertension; PAH–pulmonary arterial hypertension; CTD–connective tissue disease; CHD–congenital heart disease; CTEPH–chronic thrombembolic pulmonary hypertension.

When compared to the rest of PH group, patients with PAH associated with congenital heart disease (CHD) differed significantly in age (41 ± 17 years), prevalence of arterial hypertension (17%) and diabetes mellitus (13%), value of PAMP (70 ± 23 mm Hg), TAPSE (16 ± 5 mm), distance during 6MWT (382 ± 114 m), follow-up period (5.8 ± 2.7 years) and mortality rate (25%). Patients with PAH associated with connective tissue disease (CTD) differed from other subgroups in the right atria (RA) and right ventricle (RV) size (42 ± 8 mm and 40 ± 8 mm), advanced NYHA class (95% were in NYHA III or IV) and in a higher occurrence in the male gender (22%). More details are in S1 Table.

### Prevalence of SVT in different subgroups and according to etiology

The prevalence of SVT in the Ipc-PH group was 25% (n = 162). The details of the spectrum of the first documented arrhythmia are listed in Table 3.

**Table 3:** First documented supraventricular tachycardia in isolated pre-capillary pulmonary hypertension.

Clinical classification of PH	Total n = 641	Group 1 PH			Group 3 PH	Group 4 PH	Group 5 PH	P value
		PAH Idiopathic/heritable n = 220	PAH associated with CTD n = 62	PAH associated with CHD n = 52	Lung disease/hypoxia n = 140	CTEPH No PEA n = 130	Unclear/Multifactorial n = 37	
**No of patients with SVT**	162 (25%)	54 (25%)	18 (29%)	18 (35%)	23 (16%)	39 (30%)	10 (27%)	0.01
**Typical AFL**	28 (4.4%)	12 (5.5%)	2 (3.2%)	3 (5.8%)	1 (0.7%)	8 (6.2%)	2 (5.4%)	NS
**AF total**	110 (17%)	34 (16%)	16 (26%)	9 (17%)	20 (14%)	25 (19%)	6 (16%)	0.01
**AF paroxysmal**	49 (7.6%)	9 (4.0%)	10 (16%)	4 (7.7%)	11 (7.8%)	13 (10%)	2 (5.4%)	NS
**AF persistent**	23 (3.6%)	9 (4.0%)	2 (3.2%)	2 (3.8%)	1 (0.7%)	6 (4.6%)	3 (8.1%)	NS
**AF permanent**	38 (5.9%)	16 (7.3%)	4 (6.4%)	3 (5.8%)	8 (5.7%)	6 (4.6%)	1 (2.7%)	NS
**AVNRT**	4 (0.6%)	1 (0.5%)	0	1 (1.9%)	0	1 (0.8%)	1 (2.7%)	NS
**AVRT**	0	0	0	0	0	0	0	-
**Focal AT**	1 (0.2%)	0	0	1 (1.9%)	0	0	0	-
**Other AT**	19 (3.0%)	7 (3.2%)	0	4 (7.7%)	2 (1.4%)	5 (3.8%)	1 (2.7%)	NS
**Proportion of patients with SVT onset prior to diagnosis of PH**	51 (8.0%)	13 (5.9%)	8 (13%)	5 (9.6%)	7 (5%)	14 (11%)	4 (11%)	NS
**-at diagnosis of SVT and PH**	49 (7.6%)	14 (6.4%)	6 (9.7%)	6 (12%)	6 (4.3%)	14 (11%)	3 (8.1%)	NS
**- after diagnosis of PH**	62 (9.7%)	27 (12%)	4 (6.5%)	7 (13%)	10 (7.1%)	11 (8.5%)	3 (8.1%)	NS

Values are expressed as n (%). P-value shows differences among major classes. NS–non-significant. PH–pulmonary hypertension; PAH–pulmonary arterial hypertension; CTD–connective tissue disease; CHD–congenital heart disease; CTEPH–chronic thrombembolic pulmonary hypertension; AFL–atrial flutter; AF–atrial fibrillation; AVNRT–atrio-ventricular nodal reentry tachycardia; AVRT–atrio-ventricular reentry tachycardia; AT–atrial tachycardia.

The most prevalent arrhythmias were AF followed by typical AFL (17% and 4.4% of all Ipc-PH patients). An excessive prevalence of SVT was found in patients with PAH associated with CHD (35%; p = 0.01). Out of all Ipc-PH patients, subjects with PAH associated with CTD had the highest incidence proportion of AF (26%; p = 0.01), Table 3. When analysing only patients with SVT, the highest proportion of AF was documented in patients with PAH associated with CTD and with PH due to lung disease (89% and 87%, p = 0.01).

**Fig 1:**
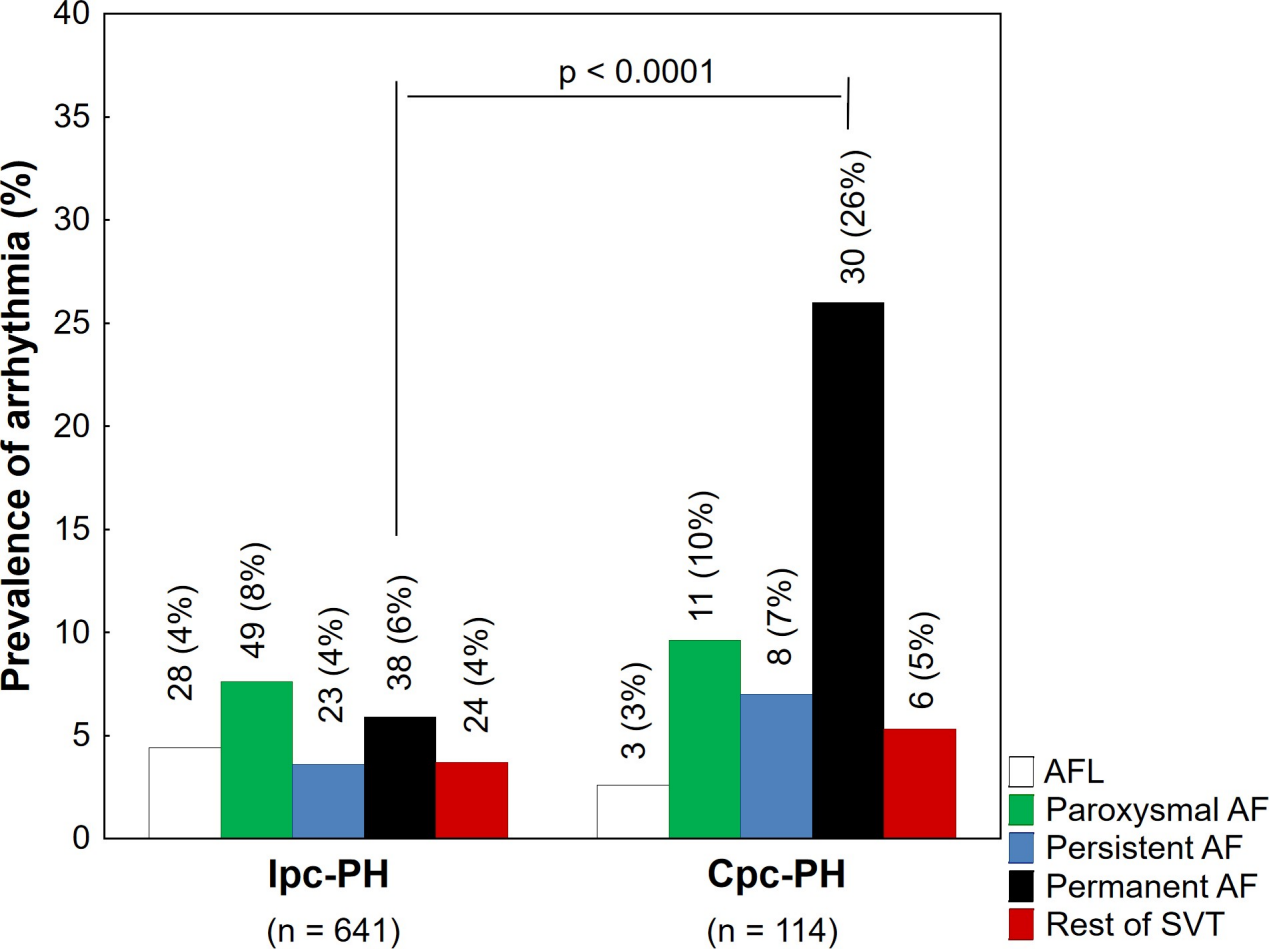
The prevalence of different supraventricular tachycardias in subgroups. **Legend:** Values are expressed as n (%). Ipc-PH–isolated pre-capillary pulmonary hypertension; Cpc-PH–combined post- and pre-capillary pulmonary hypertension; PH–pulmonary hypertension; AF–atrial fibrillation; AFL–atrial flutter; SVT–supraventricular tachycardia.

Out of all patients with SVT, arrhythmia was detected before diagnosis in 51 (31%) cases. In 49 (25%) patients the diagnosis of PH and SVT were made at same time. The rest of patients (62 (38%)) manifested SVT after the diagnosis of PH during a follow-up, Table 3. Out of all patients with Cpc-PH (n = 114), 58 (51%) subjects had SVT. The prevalence of SVT in this group was higher compared to Ipc-PH (51% vs. 25%, p < 0.0001). This difference is mostly given by the higher occurrence of permanent AF in the Cpc-PH group (Fig 1).

**Fig 2:**
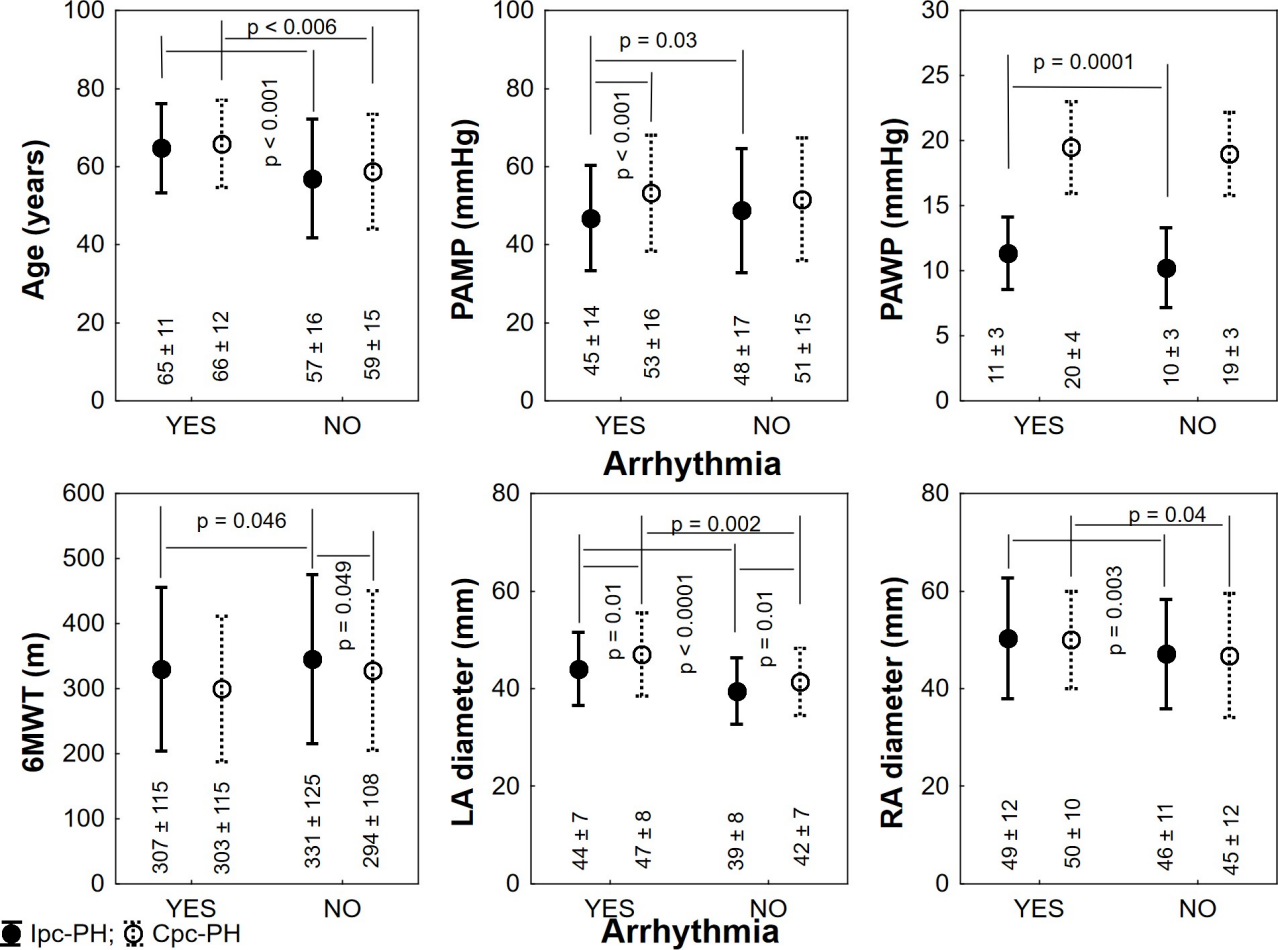
Differences between patients with and without supraventricular tachycardia in relation to presence of post-capillary component. **Legend:** Values are expressed as mean ± standard deviation or as n (%). Ipc-PH–isolated pre-capillary pulmonary hypertension; Cpc-PH–combined post- and pre-capillary pulmonary hypertension; PAMP–pulmonary arterial mean pressure; PAWP–pulmonary arterial wedge pressure; 6MWT–six-minute walking test; LA–left atrium; RA–right atrium.

Fig 2 shows the clinical profile of patients with Ipc-PH and Cpc-PH with or without arrhythmia. A comparison of the two groups focusing on patients with SVT has shown that Cpc-PH had a higher PAMP and larger LA dilatation. There was no difference in left ventricular size or systolic function in SVT patients with Cpc-PH and Ipc-PH. By the final follow-up, 39 (67%) Cpc-PH patients with SVT and 88 (54%) patients with Ipc-PH and SVT had died; p = 0.09 (Gehan-Wilcoxon test).

### Treatment strategies in patients with PH developing SVT

Out of all SVT patients (n = 220), rhythm control strategy (including both pharmacological and non-pharmacological methods) was used in 143 (65%). Catheter ablation (CA) was used in all patients with AVNRT, AVRT and focal AT with a 100% success rate. In patients with AFL, a CA was performed in 22 (71%) patients. Rhythm control drug treatment with amiodarone was used in 4 (13%) cases. The rest of the patients (5 (16%)) were treated by betablockers. At the final follow-up, sustained SR was present in 27 (86%) of all the subjects with AFL. In AF and related AT, rhythm control was attempted in 64 (40%) patients. A non-pharmacological strategy (i.e. CA) was applied in 7 (5%) patients. Amiodarone and propafenone was administered in 39 (22%) and 13 (7%) patients. 5 (3%) subjects were treated with sotalol. The rest of AF patients were on betablockers (69 (38%)), verapamil (16 (9%)) or digoxin (11 (6%)) or stayed without antiarrhythmic treatment (20 (11%)). Despite rhythm control strategy (both pharmacological and non-pharmacological), the recurrence rate of AF and related AT was 21%.

Out of 211 patients with arrhythmias with an increased thromboembolic risk (AVNRT, AVRT or focal AT were excluded), 182 (86%) patients were anticoagulated. Majority of anticoagulated patients was on vitamin K antagonists (150, 71%). Antiplatelet therapy with acetylsalicylic acid was used for 20 patients (9%).

### Clinical predictors of mortality in overall PH population

By the time of the final follow-up, 415 (55%) patients died. The mean time interval from diagnosis to death was 2.7 ± 2.2 years. In the rest of the patients, the mean follow-up was 5.2 ± 2.7 years. According to univariate analysis patients over 60 years of age, males, subjects with NYHA ≥ III class, lower 6MWT distance, higher RAP, higher RA diameter and Cpc-PH were more likely to die, Table 4.

**Table 4:** Clinical outcome.

	Univariate analysis			Multivariate Cox regression analysis		
	Dead NO N = 340	Dead YES N = 415	p	RR	95% CI	p
**Prevalent SVT**	93 (27%)	127 (31%)	NS	-	-	-
**Prevalence AF**	66 (19%)	93 (22%)	NS	-	-	-
**Age < 60 years**	171 (50%)	132 (32%)	<0.001	0.49	0.38–0.65	<0.001
**Male gender**	118 (35%)	191 (46%)	0.002	1.48	1.18–1.87	<0.001
**Arterial hypertension**	191 (56%)	243 (59%)	NS	-	-	-
**Diabetes mellitus**	89 (26%)	126 (30%)	NS	-	-	-
**Stroke or Systemic embolism**	23 (7%)	33 (8%)	NS	**-**	**-**	**-**
**NYHA III or IV classes**	225 (66%)	382 (92%)	<0.001	1.77	1.19–2.65	0.005
**6MWT < 350 m**	119 (35%)	255 (61%)	<0.001	1.88	1.42–2.49	<0.001
**RAP < 10 mm Hg**	188 (55%)	179 (43%)	0.001	1.02	0.80–1.29	0.54
**PAMP < 50 mm Hg**	214 (63%)	238 (57%)	NS	-	-	-
**LA diameter < 40 mm**	164 (48%)	167 (40%)	NS	-	-	-
**RA diameter < 48 mm**	221 (65%)	179 (43%)	<0.001	0.67	0.54–0.86	0.001
**Cpc-PH**	40 (12%)	74 (18%)	0.023	1.22	0.90–1.66	0.20

Values are expressed as n (%). NS–non-significant. SVT–supraventricular tachycardia; AF–atrial fibrillation; SR–sinus rhythm; 6MWT–six-minute walking test; RAP–right atrial pressure; PAMP–pulmonary arterial mean pressure; LA–left atrium; RA–right atrium. Cpc-PH–combined post- and pre-capillary pulmonary hypertension.

In a multivariate cox regression model, independent predictors of mortality included advanced age, male gender, NYHA ≥ III class, reduced 6MWT distance and increased RA diameter, Table 4. The prevalence of SVT, prevalence of AF or absence of a stable SR at the end of the study period were not identified as mortality predictors in any tested model.

## Discussion

Our data shows a high prevalence of SVT in a relatively large, unselected and real-world population of patients with PH. The major finding of this study is that the prevalence of arrhythmias is not the same in different PH classes. The prevalence of SVT was substantially higher in patients Cpc-PH as compared to Ipc-PH mainly due to an increased occurrence of persistent and permanent forms of AF. The results confirm an association of SVT with clinical deterioration in overall PH patients. Our data does not identify SVT as an important independent predictor of mortality.

### Pathophysiological mechanism of SVT in PH

Apart from clear mechanisms of SVT (i.e. AVNRT, AVRT or common AFL, triggers of AF in pulmonary veins), an arrhythmogenic mechanism of complex atrial tachycardias including AF in the population of PH patients appears to be more complicated. There is evidence supporting the right atrial substrate for complex atrial arrhythmia: PH leads to an upstream enlargement of the RA as consequence of RV overload and functional impairment [18]. A long-standing PH is associated with progressively slowing conduction, reduced tissue voltage and regions of electrical silence in the RA [19]. Finally, modulations of the autonomic system together with an elevated right heart filling pressure may trigger and perpetuate related arrhythmia [20,21].

Recently, we have shown, that the left sided substrate could play a role in arrhythmogenesis of complex atrial arrhythmia, even in isolated precapillary PH [8].

### Comparison to previous reports

The overall prevalence of SVT in our large PH population was slightly higher than in previous studies. Most retrospective and several prospective studies have reported a cumulative incidence of SVT ranging from 10 to 25% of patients with PAH or inoperable CTEPH [6,7,11,12]. The prevalence is still high, even if the Cpc-PH group is analysed separately. The higher prevalence of SVT in the overall PH groups in our study is more likely due to applied inclusion criteria. According to our protocol, all detected SVTs in a patient’s entire personal history were taken into the account. Thus, our data refers to cumulative prevalent cases including baseline and new incident cases together. The second explanation for the relatively higher incidence of SVT in Ipc-PH in our study might be given by potential overlap between groups. Some patients in Ipc-PH group may actually have Cpc-PH, which is characterised by an increasing incidence of SVT. In our study, a significant proportion of patients with Ipc-PH had risk factors for heart failure with preserved ejection fraction, i.e. arterial hypertension or diabetes mellitus. As suggested by Opitz [22], these cases represent a borderline category of patients with “atypical idiopathic PAH” in whom left heart involvement remains silent under resting conditions. PAH diagnosis is based uniquely on resting invasive pulmonary pressure measurements. It has been repeatedly shown [23,24] that a fluid challenge or exercise can unmask a postcapillary component in a large number of patients. This may lead to a diagnosis of purely precapillary PAH in a group of patients, in whom pulmonary hypertension is actually a Cpc-PH.

The most prevalent arrhythmias in our cohort were AF and AFL. This finding is in agreement with previous studies [3,5–7,9]. However, some series indicated that AF and AFL were equally common [5,7,9]. The excessive AF prevalence in our study could be explained by regular follow-ups focusing more on rhythm disorders that improved the detection of both symptomatic and silent persistent AF. The high AF prevalence could also be a consequence of including more potentially dormant Cpc-PH to the study.

### Prevalence of arrhythmia in different PH subgroups

In Ipc-PH patients we observed the highest occurrence of all SVTs (mostly AF) among patients with PAH associated with CHD. We speculate, that the manifestation of the most advanced impairment of the right heart and the highest values of PAMP together with the long duration of the disease are most likely responsible for the high arrhythmia prevalence in the PAH associated with CHD.

The highest proportion of AF was identified among patients with PAH associated with CTD. We hypothesize, that other mechanisms over PH must be proarrhythmogenic in CTD. Chronic inflammation and treatment (i.e. corticosteroids) in those patients with CTD could play a significant role [25]. In relative numbers, the excessive incidence of AF was also documented in subjects with hypoxic PH. This observation is well in line with a previous study that showed a higher prevalence of arrhythmogenic foci arising from the RA in patients with chronic lung disease [26].

### Comparison of arrhythmia occurrence in patients with Ipc-PH and Cpc-PH

When a post-capillary component of PH is present, the mechanisms of arrhythmia are most likely different from Ipc-PH [11] and correspond more likely to proarrhythmogenic substrate in left heart disease. It is known that the prevalence of atrial arrhythmias (especially AF) has been from 10% to 40% in the left ventricular disease [27–29]. In patients with PH due to left heart disease, AF is prevalent in almost 60% [11]. Therefore, it is not surprising that the increase of the prevalence of SVT in the Cpc-PH group is mainly due to a higher occurrence of persistent or permanent AF. The high burden of persistent or permanent arrhythmia may be a result of LA remodeling [30,31] caused by the increased left atrial pressures documented by the elevated PAWP. On the other hand, patients with AF have frequently decreased atrial contraction, atrio-ventricular asynchrony, and a rapid heart rate with a reduction of diastolic filling which may represent the cause of an increase in PAWP [28,32–35]. Of note, patients with Cpc-PH and SVT had more severe PH, worse functional capacity and an increased mortality rate, as compared to Ipc-PH patients with SVT.

When compared to the study performed by Rottlaender including post-capillary PH [11], we observed a lower cumulative incidence of arrhythmias (51% for all SVT and 43% of AF) in Cpc-PH. This finding may be explained by the fact that our population did not include cases with severe left heart failure and all patients with isolated post-capillary PH were a priori excluded from the analysis. In our study, the Cpc-PH population had only small rise in PAWP (maximum 25 mm Hg) over prevalent pre-capillary component.

### Clinical outcome of SVT

Our data is in line with previous studies reporting that SVT in PH patients is associated with functional deterioration and/or right ventricular failure [3,5–7,9,18]. Olsson et al. identified that the estimated survival rate after the diagnosis of a PH was reduced in patients with permanent AF compared to patients with transient episodes or without arrhythmia [5]. Another study confirmed that SVT in patients with idiopathic PAH presages substantial morbidity and mortality and may be a determinant of mortality [3]. In our study however, rather than SVT manifestation, advanced age, male gender, deterioration of functional parameters and a larger RA diameter, were stronger predictors of mortality in a multivariate analysis. Several explanations may be offered. The first component is more likely given to the inclusion criteria applied. According to our protocol, all detected SVTs in the entire personal history were taken into the account. Thus, our data refers to a cumulative prevalence including clinically silent, accidentally diagnosed cases and cases with clear clinical deterioration together. Next, the lack of regular monitoring did not allow for identifying the exact arrhythmia burden. Real arrhythmia burden might bear a higher weight than the history of SVT in an analysis of the clinical outcome. We also speculate, that this finding is more likely based on precise management of SVT, especially AF, in the cardiological department including anticoagulation. Despite lack of evidence, we also hypothesise that modern rhythm control strategy could improve the clinical outcome in some patients with advanced PH. The number of patients treated with antiarrhythmic drugs or indicated for a catheter ablation could warrant this hypothesis. On the other hand, our study was not focused on testing the best treatment strategy and its design did not allow for comparing rate and rhythm control.

There are some other markers of severe PH and clinical deterioration, i.e. duration of QRS complex or QTc interval, which were not analysed in our study. It has been shown that prolongation of QRS and QTc was associated with severity of PH and worse clinical outcome [36,37].

### Limitations

Our study has several limitations including the use of information from single centre, its retrospective design and absence of regular and systematic rhythm monitoring. The data was based on standard electrocardiograms and carefully analyzing a patient's history. Because of the lack of other means of rhythm monitoring performed on regular basis, it is likely that some self-terminating, clinically silent SVT episodes might have been missed. However, in all previously published retrospective and prospective studies, the prevalence of SVT in patients with PH was based on a standard ECG. We expect that periodic and prolonged ECG monitoring would provide more realistic estimates on the prevalence of SVT. We speculate that the true prevalence of SVT might be higher than reported. The second limitation is a discrepancy in the time point of collected baseline clinical variables and SVT manifestation. Clinical variables, including age, functional capacity or haemodynamics, were mostly set at time of PH diagnosis. However a significant number of SVT manifested before or later during a follow-up. The third limitation of the study was the smaller number of cases in the Cpc-PH group. The fourth limitation is that the results cannot be translated to a general population of Cpc-PH patients. The study Cpc-PH population had only relatively small rise in PAWP. The fifth limitation is that some overlap might exists between Ipc-PH and Cpc-PH patients. The diagnosis of Ipc-PH was uniquely on resting invasive pulmonary pressure measurements. The last limitation was the absence of a control group. The results are not able to identify an optimal treatment strategy for SVT in patients with different types of PH.

## Conclusions

Our study identifies that different spectrum and prevalence of arrhythmia might be expected in different etiologies of PH. An excessive number of persistent or permanent AF is responsible for an increased prevalence of arrhythmia in subjects with present Cpc-PH. Patients with borderline PAWP values and a manifestation of AF should be tested more to exclude combined PH. In the overall PH population, a history of any SVT and heart rhythm at the end follow-up was not associated with mortality. More prospective and multicentre studies must be done to identify optimal management of patients with arrhythmia and different types of PH.

## Supporting information

S1 Table Baseline demographic and clinical characteristics according to aetiology of PH. (DOCX)
