# Supplementary material for: The prevalence and clinical outcome of supraventricular tachycardia in different etiologies of pulmonary hypertension
Source: PLoS One. 2021 Jan 20;16(1):e0245752. doi: 10.1371/journal.pone.0245752 (PMC7817034; doi:10.1371/journal.pone.0245752)
Supplement: S1 Table — (DOCX) [file pone.0245752.s001.docx]

**S1 Table** Baseline demographic and clinical characteristics according to aetiology of PH.

| **Clinical classification of PH** | **Group 1 PH** | | | **Group 3 PH** | **Group 4 PH** | **Group 5 PH** | **p**  **(ANOVA)** |
| --- | --- | --- | --- | --- | --- | --- | --- |
|  | **PAH**  **Idiopathic / heritable** | **PAH associated with CTD** | **PAH associated with CHD** | **Lung disease / hypoxia** | **CTEPH** | **Unclear / Multifactorial** |  |
| **n** | 220 | 62 | 52 | 140 | 130 | 37 | - |
| **Age (years)** | 59 ± 16 | 60 ± 14 | 41 ± 17 | 62 ± 9 | 66 ± 12 | 61 ± 11 | < 0.0001 |
| **Males** | 100 (39%) | 15 (22%) | 19 (30%) | 85 (53%) | 71 (45%) | 17 (37%) | < 0.0001 |
| **Art. hypertension** | 154 (60%) | 34 (50%) | 11 (17%) | 99 (61%) | 105 (66%) | 28 (61%) | < 0.0001 |
| **Stroke / Systemic embolism** | 18 (7%) | 5 (7%) | 7 (11%) | 10 (6%) | 12 (8%) | 4 (9%) | NS |
| **Diabetes mellitus** | 93 (36%) | 12 (18%) | 8 (13%) | 58 (36%) | 33 (21%) | 11 (24%) | < 0.0001 |
| **Specific therapy** | 222 (86%) | 53 (78%) | 55 (86%) | 59 (37%) | 61 (39%) | 25 (54%) | < 0.0001 |
| **NYHA class** |  |  |  |  |  |  |  |
| - **I** | 2 (1%) | 0 (0%) | 1 (2%) | 1 (1%) | 2 (2%) | 0 (0%) | NS |
| - **II** | 36 (16%) | 3 (5%) | 16 (31%) | 29 (21%) | 28 (22%) | 14 (38%) | < 0.01 |
| - **III** | 141 (64%) | 51 (82%) | 31 (60%) | 85 (61%) | 89 (68%) | 20 (54%) | < 0.01 |
| - **IV** | 41 (19%) | 8 (13%) | 4 (8%) | 24 (17%) | 10 (8%) | 3 (8%) | < 0.01 |
| **6MWT (meters)** | 322 ± 124 | 288 ± 106 | 382 ± 114 | 288 ± 107 | 327 ± 128 | 349 ± 111 | < 0.001 |
| **LA in PLAX (mm)** | 42 ± 8 | 41 ± 8 | 38 ± 9 | 42 ± 7 | 41 ± 7 | 43 ± 7 | < 0.01 |
| **LVEDD in PLAX (mm)** | 44 ± 8 | 46 ± 7 | 45 ± 10 | 46 ± 8 | 45 ± 8 | 49 ± 6 | < 0.01 |
| **RA in A4C (mm)** | 48 ± 10 | 42 ± 8 | 50 ± 11 | 46 ± 11 | 46 ± 11 | 46 ± 10 | < 0.0001 |
| **RV in A4C (mm)** | 45 ± 10 | 40 ± 8 | 45 ± 11 | 44 ± 11 | 43 ± 9 | 42 ± 10 | < 0.0001 |
| **TAPSE (mm)** | 18 ± 5 | 19 ± 5 | 16 ± 5 | 19 ± 5 | 19 ± 5 | 21 ± 7 | < 0.0001 |
| **PAMP (mmHg)** | 50 ± 14 | 43 ± 13 | 70 ± 23 | 42 ± 13 | 45 ± 12 | 41 ± 15 | < 0.0001 |
| **PAWP (mmHg)** | 12 ± 5 | 11 ± 4 | 13 ± 5 | 12 ± 4 | 13 ± 4 | 13 ± 6 | NS |
| **RAP (mmHg)** | 11 ± 6 | 9 ± 5 | 9 ± 4 | 10 ± 6 | 11 ± 6 | 10 ± 6 | NS |
| **Follow-up (years)** | 4.3 ± 2.8 | 3.9 ± 3.1 | 5.8 ± 2.7 | 2.4 ± 2.2 | 3.8 ± 2.7 | 3.4 ± 2.4 | < 0.0001 |
| **Dead** | 151 (58%) | 47 (69%) | 16 (25%) | 106 (66%) | 68 (43%) | 27 (59%) | < 0.0001 |
| **SVT prevalence** | 77 (30%) | 21 (31%) | 27 (42%) | 31 (19%) | 49 (31%) | 15 (33%) | < 0.01 |

**Legend:** Values are expressed as mean ± standard deviation or as n (%). P-value shows differences among major classes. NS – non-significant. PH – pulmonary hypertension; PAH – pulmonary arterial hypertension; CTD – connective tissue disease; CHD – congenital heart disease; CTEPH – chronic thrombembolic pulmonary hypertension; 6MWT – six minute walking test; LA – left atrium; RA – right atrium; RV – right ventricle; TAPSE – tricuspid annular plane systolic excursion; PAMP – pulmonary arterial mean pressure; PAWP – pulmonary arterial wedge pressure; RAP – right atrial pressure; Cpc-PH - combined post- and pre-capillary PH; SVT – supraventricular tachycardia; PLAX – parasternal long axis view; A4C – apical four chamber view.
